# Antagomirs drive diverging DNA damage repair and stress responses in spine versus eyes during simulated spaceflight

**DOI:** 10.1016/j.bbrep.2026.102865

**Published:** 2026-11-10

**Authors:** Nicole E. Bodi, Ryan C. Miller, Shahna S. Hameed, Angela J. Kubik, Noah G. Allen, Selin Altinok, Leah Oswalt, Rebekah Sanchez-Hodge, Jonathan C. Schisler, Elizabeth A. Blaber, Afshin Beheshti, Tasneem P. Sharma

**Affiliations:** a Department of Biochemistry, Molecular Biology, and Pharmacology, Indiana University School of Medicine, Indianapolis, IN, 46202, USA; b University of Vermont, 111 Colchester Avenue, Burlington, VT, 05401, USA; c Eugene and Marilyn Glick Eye Institute, Department of Ophthalmology, Indiana University School of Medicine, Indianapolis, IN, 46202, USA; d Department of Biomedical Engineering, Shirley Ann Jackson Center for Biotechnology & Interdisciplinary Studies, Rensselaer Polytechnic Institute, Troy, NY, 12180, USA; e McAllister Heart Institute, Department of Pharmacology, And Department of Pathology and Lab Medicine, The University of North Carolina at Chapel Hill, Chapel Hill, NC, 27599, USA; f Center for Space Biomedicine, Trivedi Institute for Space and Global Biomedicine, University of Pittsburgh School of Medicine, Pittsburgh, PA, USA; g Computational and Systems Biology, University of Pittsburgh School of Medicine, Pittsburgh, PA, USA; h McGowan Institute for Regenerative Medicine, Department of Surgery, University of Pittsburgh School of Medicine, Pittsburgh, PA, 15219, USA; i Department of Bioengineering, University of Pittsburgh, Pittsburgh, PA, USA; j Broad Institute of Massachusetts Institute of Technology and Harvard, Cambridge, MA, 02142, USA

**Keywords:** MicroRNAs, Antagomirs, Galactic cosmic radiation, Hindlimb unloading, DNA damage repair response, Mitochondrial genes

## Abstract

Spaceflight is associated with systemic effects, including central nervous system syndromes. To understand the effects from spaceflight syndromes, mouse spinal and ocular tissue were exposed to experimentally calculated galactic cosmic radiation and microgravity simulations. A cohort was also treated with antagomirs for let-7a-5p, miR-125b-5p, and miR-16-5p. In spinal tissue, there was altered expression of DNA damage repair, mitochondrial, and neurodegenerative genes in antagomir-treated groups. In ocular tissue, there were no changes in DNA damage repair, but lessened antioxidant enzyme and inflammatory gene expression in antagomir-treated groups. There were differential effects of how antagomirs modulated gene expression based on spaceflight stressor and tissue.

## Highlights

• Utilized antagomirs for let-7a-5p, miR-125b-5p, and miR-16-5p in mouse CNS tissue.
• Antagomir treatment altered stress-related genes differently based on tissue type.
• Co-simulations of microgravity and radiation with antagomirs alter gene fold change.

## Graphical abstract

**Figure:**
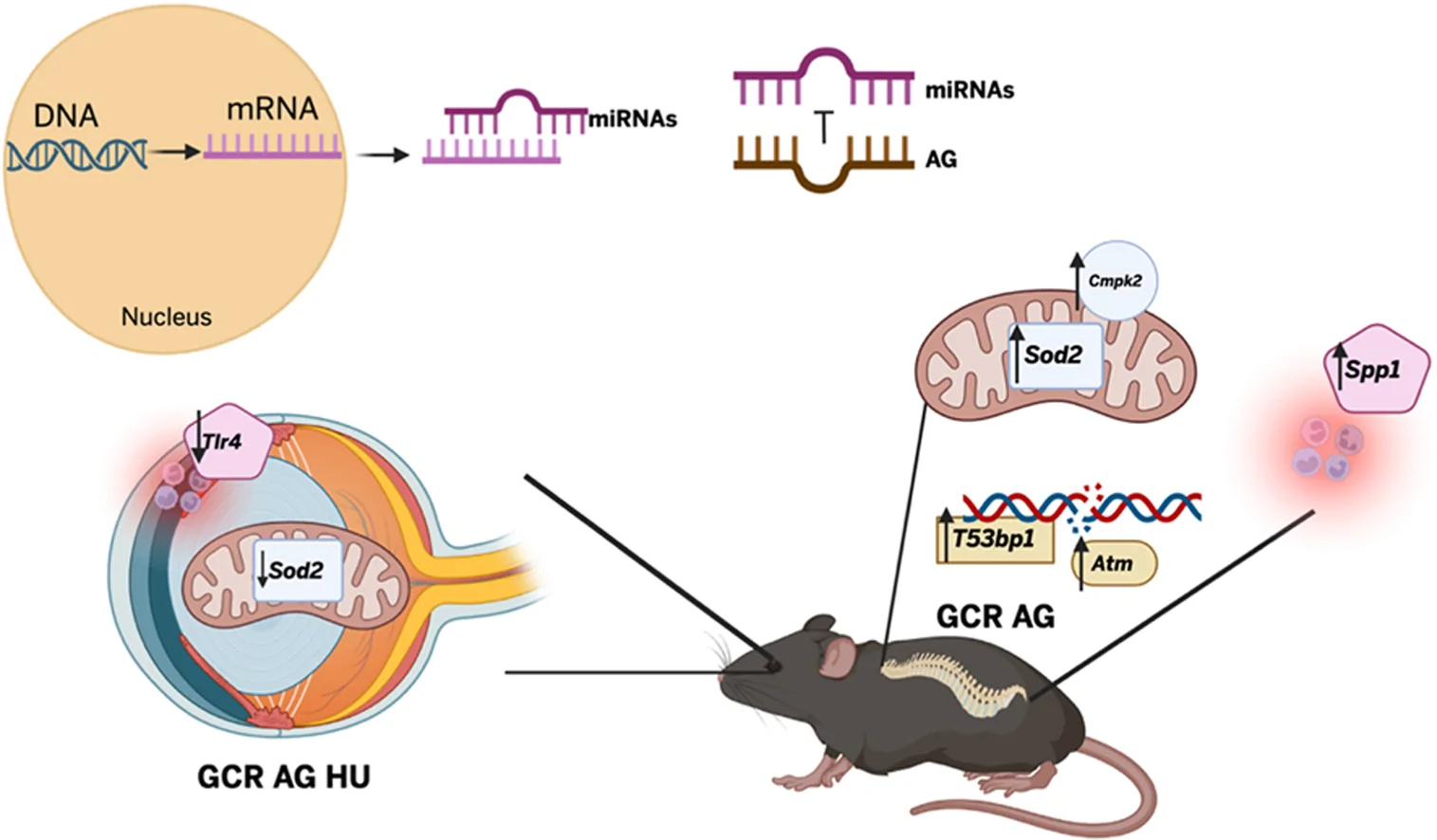

## 1 Introduction

Humans are pushing the boundaries of space exploration with the goal of sending astronauts to the Moon and on to Mars. Numerous changes occur within the body due to space travel, which include clinical ophthalmic findings due to long duration space missions [1], [2]. These are collectively referred to as spaceflight associated neuro-ocular syndrome (SANS). SANS clinical findings include optic disc edema, globe flattening, choroidal or retinal folds, focal areas of ischemic retina, hyperopic refractive error shifts, and retinal nerve fiber thickening [2], [3], [4]. These ophthalmic changes mostly resolve after time spent recovering on Earth, however some changes have been reported to persist for up to a year postflight [2], [5], [6], [7]. Since vision is a critical function of an astronaut's operational capacity, it requires mitigation, especially for long duration missions such as Mars missions where return to Earth is not readily accessible [8]. Without straightforward return to Earth, the resolution and lasting impacts of SANS clinical findings are unknown. To manage these ophthalmic changes, it is critical to elucidate the causative factors of SANS and test therapeutics.

Proposed causative factors of SANS include altered fluid dynamics that result in a cephalad fluid shift, which can lead to mildly elevated intracranial pressure (ICP). Other proposed causative factors include radiation exposure, genetic predispositions, and mitochondrial dysfunction [4], [9], [10], [11], [12], [13], [14], [15]. Mitochondrial dysfunction in spaceflight, especially in the eye, is of great interest to the space biology community. This is due to the high metabolic load of the retina and the established role between space stressors and mitochondrial changes [14], [16], [17], [18]. Therefore, to elucidate the causative factors of SANS, appropriate models must be used. Mouse models are an *in vivo* model that can act as a ground analog of spaceflight through exposure to simulated spaceflight stressors [19]. Mouse models exposed to simulated space stressors of microgravity and radiation have been reported to have systemically increased stress pathways induced by a spaceflight-associated circulating microRNA (miRNA) signatures [20]. Different miRNAs can be differentially expressed under varying conditions. A therapeutic option to mitigate cellular changes associated with spaceflight are antagomirs (AG), which inhibit the effects of miRNAs [20], [21]. There are 13 circulating miRNAs that have previously been associated with systemic changes in both rodent and human models of spaceflight [20], [22]. Furthermore, previous research performed in the retina has shown that miRNA dysregulation can disrupt normal vision and exacerbate retinal conditions [23]. However, the specific spaceflight-associated miRNA signature and resulting pathways affected in the eye are still unknown.

We have performed studies on tissue from ground analogs of spaceflight with mice exposed to simulated space stressors. These samples were exposed to a form of deep space radiation: 0.5 Gy galactic cosmic radiation (GCR) simulation [24], [25]. To simulate microgravity in mice, the hindlimb unloading (HU) method was utilized. In short, the method was developed to elevate the hindlimbs of the mice to generate a 30° head-down tilt, thus recreating the fluid shifts observed in actual spaceflight [26], [27], [28]. With these models, we aimed to investigate the impacts of spaceflight associated miRNAs within spinal and ocular tissue. The connection between the spinal cord and eye may be present as a phenomenon of altered fluid dynamics. Cerebrospinal fluid, which is within the subarachnoid space of the spinal cord, moves headward during spaceflight [29], [30]. This is believed to lead to elevated intracranial pressure (ICP), which is a theorized factor in SANS development [4].

To address the SANS phenotype, a countermeasure focused on the molecular changes can be used. In terms of SANS, the relationship between the cardiovascular system and the eye in spaceflight models has been previously established [31], [32], [33]. In brief, in microgravity conditions there is mildly elevated ICP. The decreased drainage capacity of cerebrospinal fluid in microgravity can lead to venous congestion, and ultimately elevated ICP [32]. The miRNAs let-7a-5p, miR-125b-5p, and miR-16-5p were selected from the 13 spaceflight miRNAs due to their association with cardiovascular dysregulation in spaceflight models and increased expression following GCR exposure [20], [22], [34]. Previous studies done on these miRNAs in an *in vitro* 3D microvessel model gave credence to our goal to see if these antagomirs would be beneficial in an *in vivo* model of central nervous system (CNS) tissues [20], [22]. This is due to the linkage between the microvascular environment and the eyes and spines. The 3D microvascular model is valuable to provide insight into the vasculature of the eyes, specifically the retina. The retina has a high oxygen consumption rate, in relation to the tissue unit weight, therefore, proper functioning of this vasculature is required to maintain retina health [35]. In the spine, vasculature is crucial in delivering oxygen and providing venous drainage [36], [37]. Therefore, through examining the results from 3D microvascular models that were responsive to the AG for these three miRNAs and applying them to an *in vivo* model, we can understand how AG can impact the CNS structures in response to GCR or HU stressors. Previously, let-7a, miR-125, and miR16 have been found to have connections to cancer and cell signaling [38], [39], [40], [41]. But in a human 3D microvascular model, miRNA inhibitors, AG, specific for let-7a-5p, miR-125b-5p, and miR-16-5p showed promise in preventing damage induced by GCR simulations [20], [22]. Spaceflight-specific studies have shown these AG to have the capacity to protect from GCR-induced double-stranded DNA breaks and inflammatory dysfunction via TNF-α, IL6, and interferon signaling. While additionally improving mitochondrial activity through decreasing mitochondrial dysfunction and hypoxia [22]. For our investigation with spinal and ocular tissue, we wanted to expand our understanding of DNA damage, oxidative stress, and inflammation induced by simulated spaceflight within these tissues, and evaluate how antagomirs can act as therapeutics. Through this study, we achieved an understanding of how spinal and ocular tissue differ in gene expression when treated with AG, and the potential for an ocular-specific miRNA signature that needs further investigation.

## 2 Methods

### 2.1 Animal model exposed to simulated GCR

Irradiation of animals was conducted at the NASA Space Radiation Laboratory (NSRL) at Brookhaven National Laboratory (BNL, Upton, NY). Samples were positioned in the plateau region of the Bragg curve and irradiated at room temperature. Dosimetry was performed by NSRL. Animals were irradiated with 0.5 Gy of simplified simulated GCR (referred to as GCR). This mixture was selected as it is a simplified GCR simulation and is translatable to exploratory class missions. The GCR simulation dose is an experimentally manageable and cumulative-dose approximation. However, it does not fully reproduce the chronic, low-dose-rate, and prolonged nature of a Mars mission.

To create this simulation, the irradiation used ions, energy, and doses as determined by a NASA consensus formula for 5 ions species, with protons delivered at two energies: protons at 1000 MeV, ^28^Si at 600 MeV/n, ^4^He at 250 MeV/n, ^16^O at 350 MeV/n, ^56^Fe at 600 MeV/n, and protons at 250 MeV, in the following proportions – 1000 MeV protons at 34.8%, 250 MeV protons at 39.3%, ^28^Si at 1.1%, ^4^He at 18%, ^16^O at 5.8%, and ^56^Fe at 1%. This mixture is the simplified version of the full GCR simulation and represents the proportions of ions found in space, and thus translates to exploratory class missions. The low LET particles (protons and Helium) make up the majority of charged particles while high LET ions generally have a greater relative biological effect.

### 2.2 Administration of the AG treatment to mice

AG to the following three specific miRNAs were purchased and designed from AUM biotech (Philadelphia, PA, USA) for miR-16-5p, miR-125b-5p, and let-7a-5p. Specifically, the AG were identified as AUMantagomirTM oligos. The design of the AG were created to target the entire sequence of each miRNA, which negates the capacity for these AG to bind to close family members. The AG were designed with full complementarity to the mature miRNA sequences with standard chemical modifications to enhance stability and minimize off-target hybridization. AG specific for these miRNAs have been previously used in 3D microvascular cultures [20], [22], [42]. Formerly, we have done experiments with a scrambled AG to control for non-specific effects. The scrambled antagomir produced the same impact as no antagomir/vehicle treatment [22]. For the present experiment, each AG was given at 10 mg/kg, for a combined dose of 30 mg/kg AUM AG dose in 1 x PBS by intraperitoneal (IP) injection every three days starting from one day before HU setup until one day before irradiation [43].

### 2.3 Procedure for irradiating mouse model at Brookhaven National Laboratory

All experiments were approved by BNL Institutional Animal Care and Use Committee (IACUC) (protocol number: 506) and all experiments were performed by trained personnel in AAALAC accredited animal facilities at BNL, while conforming to the U.S. National Institutes of Health Guide for the Care and Use of Laboratory Animals. All methods were carried out in accordance with the relevant guidelines and regulations and are reported in accordance with ARRIVE guidelines. Briefly, N = 120 15-week ± 3-day old, C57Bl/6J wildtype female mice were purchased from Jackson Laboratories and housed at BNL (Upton, NY). Upon arrival to BNL, mice were quarantined and acclimated to a standard 12:12 h light: dark cycle, with controlled temperature/humidity for 1-week prior to cage acclimation. Food and water were given ad libitum, and standard bedding was changed once per week. Mice were cage acclimated (N = 10 mice per group; 2 mice per cage to maintain social interaction) 3-days prior to HU, followed by 14-days either normally loaded (NL) or HU. Body weight tracking was performed on days −3, 0, 7 and 14. On day 13, mice were transported on BNL base to the NSRL facility by animal care staff and were transferred to individual HU boxes. The following doses of irradiation were administered; GCR (0.5 Gy) and Sham control (0 Gy). Mice were positioned in the plateau region of the Bragg curve and irradiated at room temperature in individual HU boxes to enable whole body irradiation while maintaining hindlimb suspension. The NSRL physics staff performed the dosimetry. This dose of radiation is equivalent to what an astronaut is predicted to receive in deep space during a Mars mission, though it is modeled as a single exposure over 25 min instead of the actual chronic exposures over 1.5 years. A 60 × 60 beam was utilized at the NSRL for irradiation. Sham controls were treated similarly to GCR irradiated mice, including HU cage boxes and beam line (without irradiation) for the same duration as GCR simulation.Irradiation was administered on day 13 and tissues were collected at 24-h post-irradiation and post-euthanasia by CO_2_ overdose, followed by cervical dislocation. The pair of eyes and the spinal cord that were used in this study were harvested from the same individual mouse. The data reflected in our results section is one of the individual eyes from the pair that had RNA extracted following harvest. The contralateral eye from the pair was paraffin fixed, but was not included in our results shown in this manuscript. All organs were flash frozen and collected at the time of dissection. All tissues were stored at −80 °C.

### 2.4 Quantitative real-time polymerase chain reaction

RNA extraction on spinal and ocular tissue was performed using the MACHEREY-NAGEL kit protocol. The RNA (100 ng) was reverse transcribed to cDNA using iScript™ kit (Bio-Rad Laboratories). TaqMan array qRT-PCR was used for assessing spinal or ocular gene expression, with reaction mixture comprised of TaqMan fast advanced master mix (Thermo Fisher Scientific, Cat. No. 4444558), cDNA, and primers in a pre-designed TaqMan array plate. The qRT-PCR was performed (Applied Biosystems QuantStudio3 system) and fold changes (FC) were calculated using 2^−ΔΔct^ with *Gapdh* as the housekeeping control.

### 2.5 Analysis of prior research complied on the NASA Open Science Data Repository (OSDR)

We consulted the NASA OSDR database to compare our findings with other experimental data. In our search, we looked for keywords associated with the results from our findings. In the study for space-flown mice, we searched “eyes” and chose the work titled: “Artificial Gravity Attenuates the Transcriptomic Response to Spaceflight in the Optic Nerve and Retina -- Retina data” (OSD-758). For LDRγ, we searched for the keywords, “hindlimb unloading” and “ionizing radiation”. This resulted in a study titled: “Low dose (0.04 Gy) irradiation (LDR) and hindlimb unloading (HLU) microgravity in mice: retina transcriptomic and epigenomic data” (OSD-203). Lastly, for Radiation, AG, and HUVECs, we searched the keyword “antagomirs” and the work we selected is titled: “3D Human Umbilical Vein Endothelial Cells (HUVECs) irradiated with GCR simulated irradiation with miRNA-based countermeasures” (OSD-577). For comparisons of the studies on the heatmap, we used FC. We calculated FC using 2^−ΔΔct^ with *GAPDH*as the housekeeping control.

### 2.6 Statistics

GraphPad Prism was used for comprehensive result analysis. The qRT-PCR data was evaluated using one-way ANOVA without matching or pairing, with Tukey's multiple comparisons test. The multiple comparisons test compared the mean of each column with the means of every other column. We assumed Gaussian distribution and homogeneity of variance in the one-way ANOVAs. Statistically determined outliers were removed using the ROUT method. Biological replicates varied based on outliers and qRT-PCR amplification from each experimental group and gene. Our figures examined differences between spinal (Fig. 1, Fig. 2, Fig. 3) and ocular tissues (Fig. 4). We did separate, within tissue statistical analyses, to compare response patterns between experimental conditions. A significance threshold of *p* < 0.05 was applied to all gene expression graphs. Data are presented as mean ± SD.

**Fig. 1:**
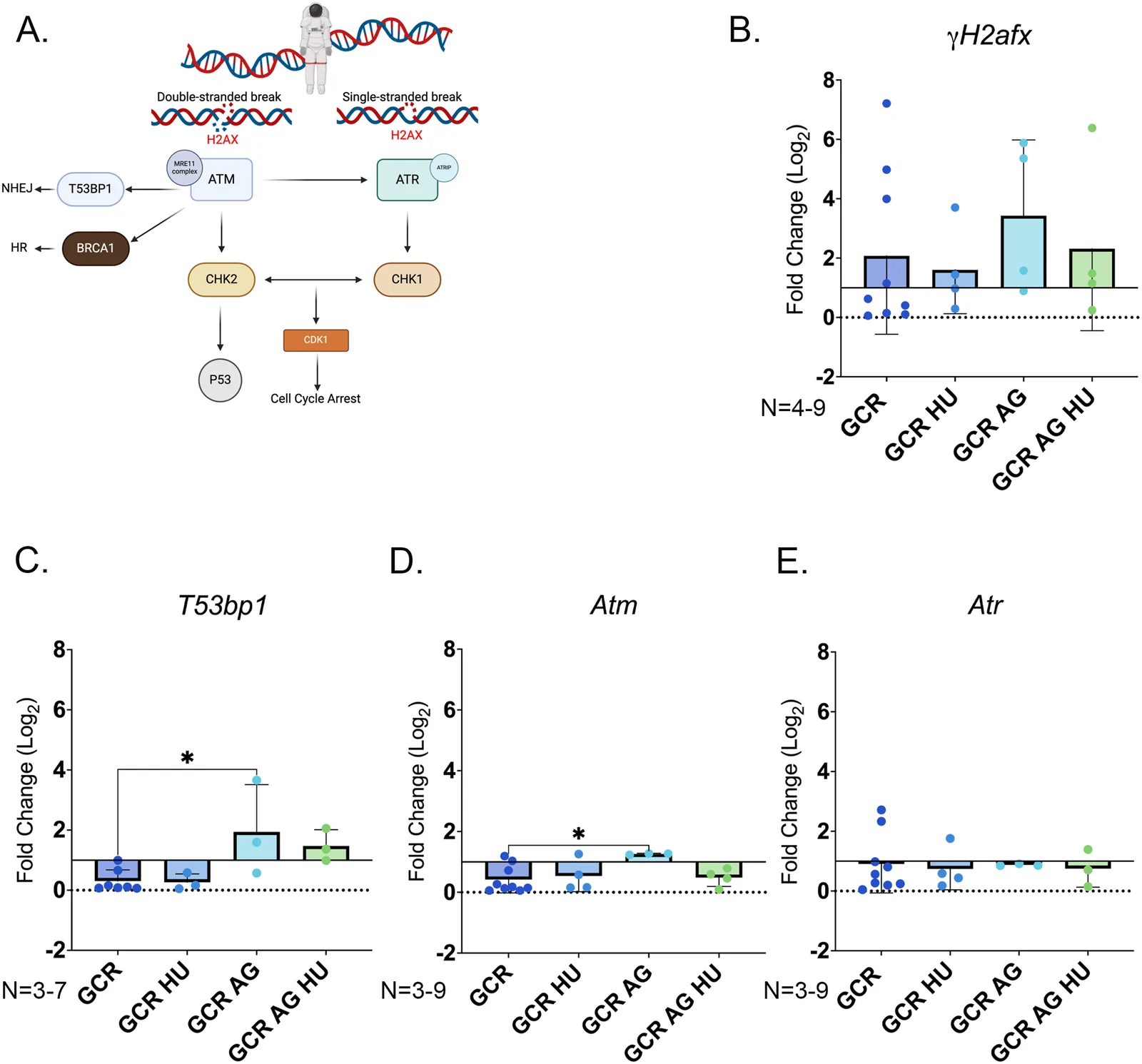
**DNA damage repair gene expression in mouse spines exposed to GCR simulations.** (A) Schematic describing DNA damage repair cycle in cells. Created with BioRender.com (https://BioRender.com/11y7k1p) (B) γ*H2afx* gene expression under galactic cosmic radiation conditions (GCR) with GCR conditions, GCR + hindlimb unloading (HU), GCR + antagomirs (AG), and GCR + AG +HU (Biological mouse replicates N = 4-9). (C) *T53bp1* gene expression in GCR, GCR + HU, GCR AG, and GCR AG HU conditions (Biological mouse replicates N = 3-7). (D) In *Atm* gene expression in GCR, GCR + HU, GCR AG, and GCR AG HU conditions (Biological mouse replicates N = 3-9). (E) *Atr* gene expression in GCR, GCR + HU, GCR AG, and GCR AG HU conditions (Biological mouse replicates N = 3-9). (Each bar represents the mean ± SD, Figures B-E one-way ANOVA, *p < 0.05).

**Fig. 2:**
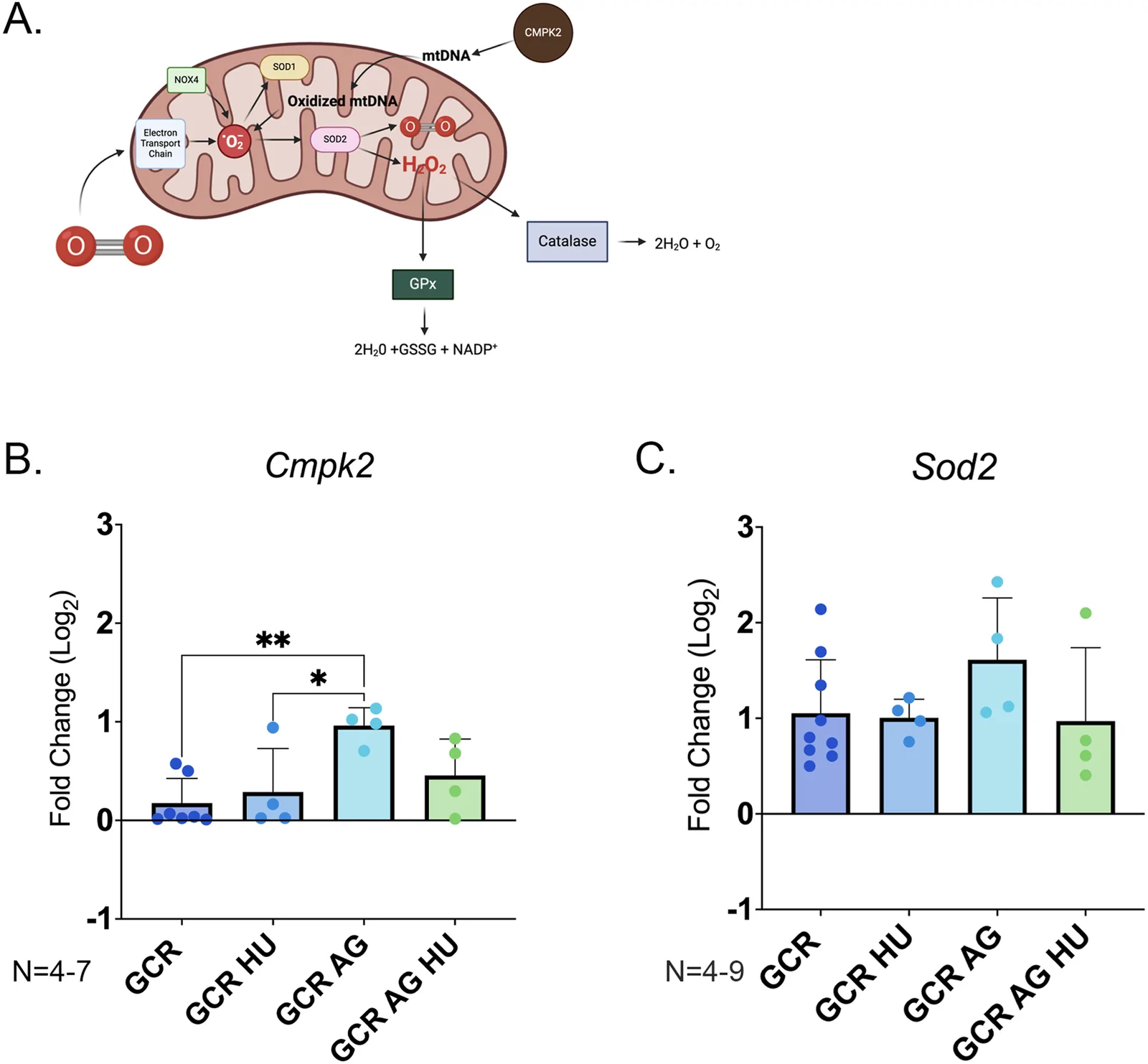
**Mitochondrial gene expression in mouse spines exposed to GCR simulations.** (A) Schematic of oxygen and reactive oxygen species (ROS) generation in the mitochondria. Created with BioRender.com (https://BioRender.com/bpmih1x) (B) *Cmpk2* gene expression in GCR, GCR + HU, GCR AG, and GCR AG HU conditions (Biological mouse replicates N = 4-7). (C) *Sod2* gene expression in GCR, GCR + HU, GCR AG, and GCR AG HU conditions (Biological mouse replicates N = 4-9). (Each bar represents the mean ± SD, Figures B-C one-way ANOVA, *p<0.05,**p < 0.01).

**Fig. 3:**
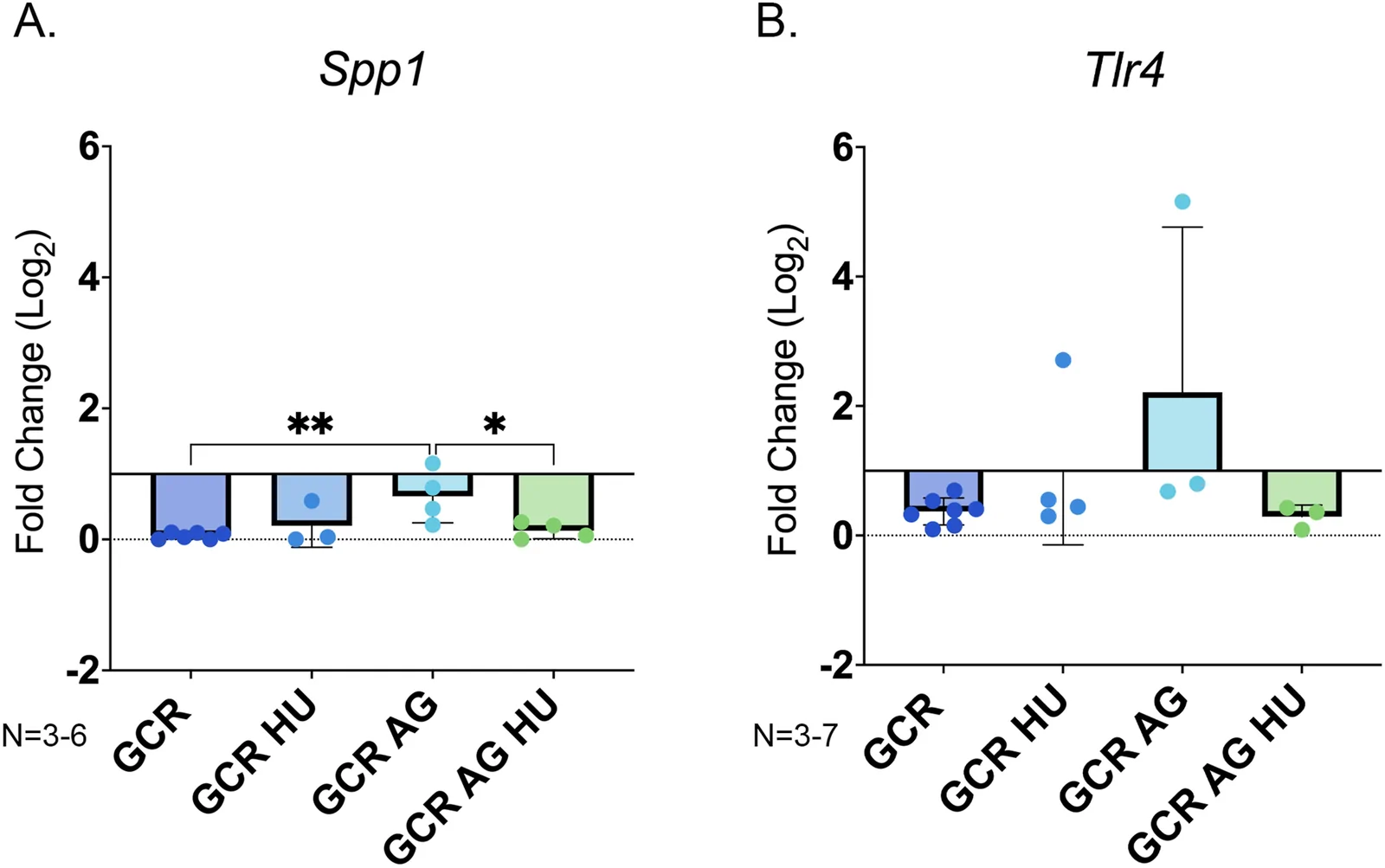
**Inflammation gene expression in mouse spines exposed to GCR simulations.** (A) *Spp1* gene expression in GCR, GCR + HU, GCR AG, and GCR AG HU conditions (Biological mouse replicates N = 3-6). (B) *Tlr4* gene expression in GCR, GCR + HU, GCR AG, GCR AG HU (Biological mouse replicates N = 3-7). (Each bar represents the mean ± SD, Figures A-B one-way ANOVA, *p < 0.05 and **p < 0.01).

**Fig. 4:**
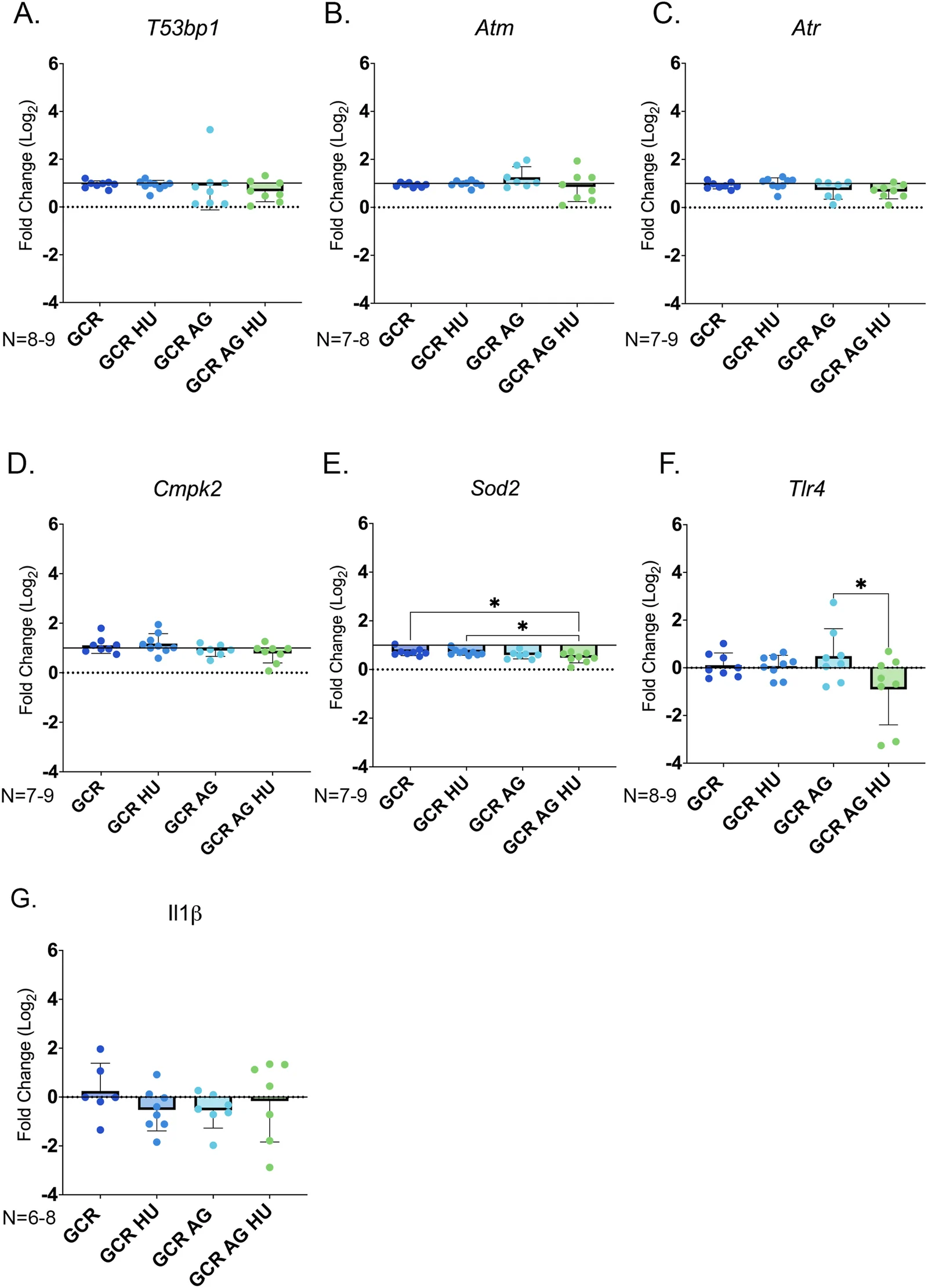
**Stress response gene expression in mouse eyes exposed to GCR simulations.** (A) *T53bp1* gene expression in GCR, GCR + HU, GCR AG, and GCR AG HU conditions (Biological mouse replicates N = 8-9). (B) *Atm* gene expression in GCR, GCR + HU, GCR AG, and GCR AG HU conditions (Biological mouse replicates N = 7-8). (C) *Atr* gene expression in GCR, GCR + HU, GCR AG, and GCR AG HU conditions (Biological mouse replicates N = 7-9). (D) *Cmpk2* gene expression in GCR, GCR + HU, GCR AG, and GCR AG HU conditions (Biological mouse replicates N = 7-9). (E) *Sod2* gene expression in GCR, GCR + HU, GCR AG, and GCR AG HU conditions (Biological mouse replicates N = 7-9). (F) *Tlr4* gene expression in GCR, GCR + HU, GCR AG, and GCR AG HU conditions (Biological mouse replicates N = 8-9). (G) *Il1*β gene expression in GCR, GCR + HU, GCR AG, and GCR AG HU conditions (Biological mouse replicates N = 6-8) (Each bar represents the mean ± SD, Figures A-G one-way ANOVA, *p < 0.05.).

## 3 Results

### 3.1 Evidence for DNA damage repair signaling, mitochondrial enzyme production, and neuroinflammation gene activity in mouse spines with AG treatment

The scope of this study was to evaluate the impact of GCR and microgravity simulations on the spinal cord and eyes. We first looked individually at the effects in spinal tissue (Fig. 1, Fig. 2, Fig. 3) and then in eyes (Fig. 4). Spinal and ocular tissues were analyzed separately using within-tissue statistical comparisons, and no direct statistical comparison between tissues was performed. This analysis was done to determine gene alterations and the impacts of AGs. The AGs were previously shown to be beneficial in reducing DNA damage repair by immunocytochemical expression in a 3D microvessel model [22]. To follow up on that finding in our models we first evaluated the impacts of DNA Damage Response (DDR) genes and pathways (Fig. 1A) in the spinal cords of the mice. After the initial source of either single-stranded or double-stranded DNA damage, *γH2afx* is localized to the site (Fig. 1A). In single-stranded DNA damage repair, the serine/threonine kinase and DNA damage sensor, *Atr*, is recruited to the site. Compared to double-stranded DNA damage, where *Atm,* which is a part of the PI3/PI4-kinase family, is recruited to the DNA damage site. Both *Atm* and *Atr* are responsible for phosphorylating downstream proteins. These include *T53bp1* which triggers non-homologous end joining (NHEJ) DNA damage repair [44]. We observed no significant changes in the γ*Hafx* gene expression under GCR, GCR + HU, GCR + AG, and GCR + AG + HU (Fig. 1B). *T53bp1* was significantly upregulated (p < 0.05, N = 3-7) in GCR AG conditions compared to GCR, but there was no change in GCR HU or GCR AG HU conditions (Fig. 1C). The *Atm* gene expression was initially downregulated in GCR and GCR HU conditions. However, *Atm* was significantly upregulated (p < 0.05, N = 3-9) in GCR AG conditions and then conversely significantly downregulated in GCR AG HU conditions (Fig. 1D). There was initially no difference in response to the initial DNA damage presence (Fig. 1B), but there was significant expression of *T53bp1* and *Atm* (Fig. 1C and D). In these figures we observed that there was upregulation of gene expression with fold change > 1 when the AG were added. However, there were no changes in *Atr* expression in either of the treated groups (Fig. 1E). Overall, we observed that there was altered NHEJ as the modality for DNA damage repair, due to the gene activity of *T53bp1* and *Atm*. Further, there was indication that AG do help with recognition of double-stranded DNA breaks, but that the addition of microgravity simulations decreases this capacity (Fig. 1C and D).

Next, we wanted to observe changes within the mitochondrial genes under our experimental conditions, specifically *Cmpk2* and *Sod2* within the spinal cord. During respiration, O_2_ enters the mitochondria and through the electron transport chain reactive oxygen species (ROS) are generated, which can be in the form of superoxide anions [45]. Additionally, *Cmpk2,* which is an enzyme that downstream leads to the production of mitochondrial nucleotide synthesis, can lead to further production of superoxide anions [46]. These ROS enter *Sod2* in the mitochondria inner membrane, to produce O_2_ and hydrogen peroxide, which are further transformed so they are no longer toxic to the cell (Fig. 2A) [47]. For *Cmpk2* (Fig. 2B), there is an initial downregulation in GCR and GCR HU conditions, but this is significantly upregulated (p < 0.01, N = 4-7) in GCR AG conditions. In *Sod2* there were no significant changes between the groups (Fig. 2C). However, although there were no significant changes, there is a trend of upregulation for *Sod2* in the GCR AG group. From the analysis of mitochondrial genes there was indication that there could be an increase in ROS generation in AG-treated groups (Fig. 2B), but that this was not present with HU. However, there were no changes in *Sod2* expression (Fig. 2C), possibly indicating that there were no changes in the response to neutralizing ROS.

The final gene panel we wanted to evaluate in the spinal cord tissue were inflammatory genes. *Spp1* is a gene involved in a variety of different areas of the body. Spinal cord specific functions are its capacity to interact with osteoclasts for bone mineralization [48]. It can also act as a responder to neurological injury, specifically in an inflammatory and neuroprotective capacity [49], [50]. In the spinal cord of our mice, we observed that *Spp1* gene expression was overall downregulated. In GCR to GCR AG, *Spp1* expression is then significantly upregulated (p < 0.01, N = 3-6), but still a fold change less than 1. There was a significant decrease (p < 0.05, N = 3-6) in GCR AG HU compared to GCR AG (Fig. 3A). The GCR AG HU group appeared to resemble the fold change value of the GCR only group. *Tlr4* is another inflammatory gene and is a responder specifically in innate immunity [51]. When investigating this gene in the spinal cord we saw that there were no significant changes in expression (Fig. 3B). We concluded that there was an indication that there was more neuroprotection, as denoted by *Spp1* expression, in the AG treated groups. Consequently, this was lost with the addition of HU. However, there were no significant changes between the groups with *Tlr4* response.

### 3.2 Evidence for antioxidant and inflammatory gene activity in mouse eyes with AG treatment

Next, we wanted to investigate the impacts of antagomir treatment to gene expression in ocular tissue of mice (Fig. 4). We first investigated the DNA damage repair pathway as previously stated (Fig. 1A). Interestingly, we saw no significant changes between the experimental conditions in *T53bp1*, *Atm*, and *Atr* (Fig. 4A–C). Next, we investigated the changes to mitochondrial genes in the mouse eyes. We saw the inverse of what we saw in the mouse spines (Fig. 2). In *Cmpk2* there were no significant changes between the experimental conditions (Fig. 4D). However, there was a significant downregulation (p < 0.05, N = 7-9) of *Sod2* in GCR AG HU compared to GCR and GCR HU conditions. There were no significant changes between GCR AG and GCR AG HU groups (Fig. 4E). This could indicate that in the eye, there is not an additional presence of ROS generated from mitochondrial DNA. However, there is a lesser demand for ROS reduction in the presence of AG and HU compared to GCR conditions alone. This is interesting due to the inverse relationship between the spinal cord tissue and the ocular tissue. Another theory, is that the radiation given while in HU, was to the dorsal part of the mouse, rather than head on. Potentially shielding the eye from direct radiation beams. However, once the ROS is in the eye, even with AG there is a lesser capacity for *Sod2* to neutralize the ROS. This could be potentially due to the additional stressor of HU, which we have shown to reverse the impacts of AG alone (Fig. 1, Fig. 2, Fig. 3). We analyzed the gene expression of *Tlr4*. There was a significant decrease in this gene in the GCR AG and GCR AG HU groups (p < 0.05, N = 8-9) (Fig. 4F). Lastly, we evaluated the gene *Il1β*, in which we saw no significant differences between the conditions (Fig. 4G). We concluded that there were little changes between the experimental groups ocular tissue, but that there is evidence for decreased *Sod2* recruitment and lesser inflammatory signature.

### 3.3 Comparing our ocular results with other experimental results

The NASA OSDR provides an opportunity for space researchers to share their raw results and publications with their colleagues. We compared the genes evaluated in ocular tissue (*Sod2*, *Cmpk2*, *Tlr4*, *Il1*β, *T53bp1*, *Atm*, and *Atr*) to their expression in other spaceflight studies (Fig. 5). These studies include *in vivo* mouse studies (space-flown mice, HU, and low dose rate (LDR) γ radiation) and an *in vitro* study (Radiation, AG, and HUVECs).

**Fig. 5:**
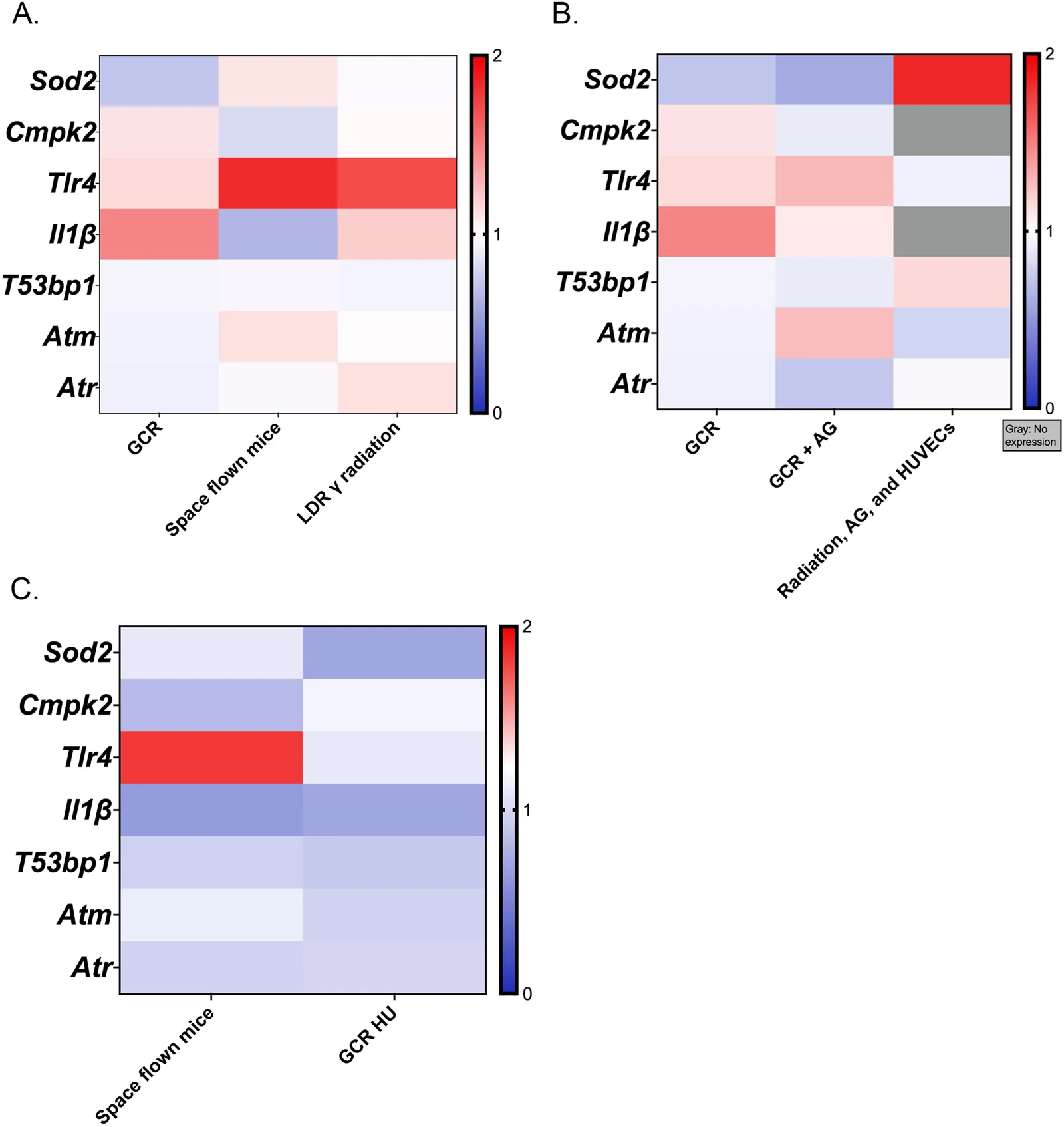
**Heatmap comparing the genes expressed in our mouse ocular tissue to the NASA Open Science Data Repository.** (A) Comparison between different types of radiation from our study and the OSDR. (B) Comparison between radiation types and treatment of AG. (C) Comparisons between actual spaceflight and simulated spaceflight to include the co-exposure to radiation and microgravity. (Statistical cutoff p < 0.05).

In the first OSDR study, we compared our high LET GCR results to data on space-flown mice and low LET LDR γ ionizing radiation. Both high and low LET have a mechanism of inducing ROS via radiolysis, meaning they will excite and ionize water molecules to create superoxide anions and secondary peroxides [52]. However, they differ in how they impact the mitochondria and response to these ROS. Low dose radiation (<0.1 Gy) can evoke regulatory mitochondrial responses eventually leading to enhanced antioxidant defense. In cases of DNA damage induced from low dose ionizing radiation, there can be a downstream increase of ATP from the mitochondria leading to DNA repair mechanisms. Meanwhile, in high LET, there is irreversible cellular and structural damage along the ionization tracks, therefore a lack of mitochondrial aid to repairing cellular components [52]. With these differences in mind, we wanted to compare high and low LET simulated space radiation to actual ISS radiation (Fig. 5A). For the space-flown mice, the experimental design was to expose mice on the international space station to centrifugation. We compared our data to the RNA sequencing data for 0 G and centrifugation-produced 1 G from the space flown mice. We observed similarities in expression from mitochondrial genes and DNA damage repair genes (*Cmpk2*, *T53bp1*, *Atm*, and *Atr*), but contrastingly there was upregulation of expression in inflammatory gene, *Tlr4*, in the space-flown mice and LDR γ radiation datasets compared to our GCR data.

The next OSDR study comparison showed the interplay between radiation and AG for *in vivo* and *in vitro* studies (Fig. 5B). We compared our data set to a study that also included AG and radiation, but in a human model system using 3D HUVECs. These cells were either untreated or treated with AG specific for miR-16-5p, miR-125b-5p, and let7a-5p which are miRNAs designed specifically for cardiovascular impacts [20], [22]. Since the radiation and AG mimicked that of our experimental paradigm, we expected to see similarities in the heatmap. We saw the starkest differences between the studies in the *Sod2*, *Cmpk2*, and *Il1*β genes. First, in *Cmpk2* and *Il1*β genes there was no reported gene expression in the Radiation, AG, and HUVECs condition in the RNA Sequencing data. In *Sod2*, we saw that in our GCR and GCR + AGthis gene was highly downregulated while in Radiation, AG, and HUVECs it was highly upregulated. Conversely, there was a mild increase in gene expression of *Il1*β in the GCR group compared to the others, with a lack of expression in the Radiation, AG, and HUVECs. In the DNA damage response gene, *T53bp1,* we observed that there was more gene expression in the *in vitro* model. There was more *Atm* gene in our GCR AG group compared to our GCR group and the OSDR AG group. In *Atr* there were overall trends of low gene expression in each of the experimental groups (Fig. 5B).

Next, we wanted to evaluate the combined impacts of microgravity and radiation in our GCR HU and the OSDR space-flown mice (Fig. 5C). We saw the most notable differences in *Sod2*, *Cmpk2*, and *Tlr4*. There was increased downregulation of *Sod2* in the GCR HU group compared to space-flown mice. The opposite was true in *Cmpk2*. *Tlr4* had clear distinctions with space flown mice having an upregulated gene expression and GCR HU having mild downregulations. The DNA damage response genes had similar expressions, which were all mildly downregulated.

## 4 Discussion

With the rise in space exploration, we need to understand the ways in which the CNS is affected by simulated spaceflight. Additionally, we need to evaluate therapies that can be used to mitigate various pathologies associated with space, including SANS. The AG used in this study were designed specifically for cardiovascular tissue, not ocular or spinal tissues, so we were interested to see if there was crosstalk in these pathways that could lead to therapeutic potential in non-cardiovascular models [20], [22]. As aforementioned there is a believed physiological link between the cardiovascular system and the eye, however, on a basic science level this still needs to be elucidated [32]. A knowledge gap is which of these 13 spaceflights circulating miRNA signatures are applicable for ocular and spinal tissue [20].

From our study, we found that in spinal tissue there were more instances of significant up-or-downregulation of genes compared to ocular tissue (Fig. 1, Fig. 2, Fig. 3). In the significant data from both the spines and eyes, we observed that there were more upregulated genes in the AG treated groups, which were then downregulated in the AG HU groups (Fig. 1, Fig. 2, Fig. 3, Fig. 4). This led us to conclude that there is a miRNA signature that is specific to microgravity that is not covered by the AG used in this experiment. Literature suggests that there are certain miRNAs that are associated with different stressors, such as microgravity [20], [22]. Microgravity has the proposed miRNA signature, let-7a-5p, let-7c-5p, miR-223-3p [20]. Furthermore, evidence of the minimal significance of gene expression in ocular tissue suggests that there is an ocular miRNA signature that was not effectively targeted by the AG used in this study (Fig. 4).

Comparisons with other space experiments also revealed some interesting variability, with *Sod2* and *Il1*β providing the most notable changes (Fig. 5). It is important to note that there are several distinctions when comparing our study to the others. First, we analyzed differing types of radiation (Fig. 5A). Overall, we observed similar gene expressions between the space-flown mice and the LDR γ radiation, which is to be expected due to these being types of ionizing radiation. In GCR conditions, we might expect to see a lesser expression of *Sod2* and *Tlr4* because the damage from high LET could be inhibiting the oxidative stress and inflammatory response [52] (Fig. 5A). In the Radiation, AG, and HUVECs there was an upregulation of an oxidative stress marker compared to our GCR and GCR AG groups (Fig. 5B). Similarities between our mouse model and the 3D microvascular model are that they were both exposed to 0.5 Gy GCRsim, however the microvascular model was exposed on culture day one, contrasting with the 17-week-old mice that were irradiated in our study. Further distinctions are the tissue source. Biological differences between mouse eyes and spine and 3D HUVECs are in their origin, proliferation, and maturity. The microvascular culture is derived from human fetal cells, making the cell type more accurately represent human heterogeneity and molecular expression of vascular tissue [53]. When addressing the gene panels between our *in vivo* mouse data and the *in vitro* human model, we can appreciate higher *Sod2.* An explanation for this is shown in literature with HUVEC models having increased *SOD2* expression [54], [55]. Therefore, we are proposing that AG have a greater protective capacity in the microvascular model compared to our mouse ocular model. The discrepancies in AG protective roles may be attributed to differences between species, emphasis on vascularization, and the modeling of endothelial dysregulation in radiation conditions. There could be a link between microvascular cultures and cardiovascular dysregulation that is associated with SANS. In our analysis of microgravity simulations, we again saw the most distinctions between *Sod2* and *Tlr4* (Fig. 5C). This phenomenon is similar to what we saw when comparing radiation types, therefore, we hypothesize that GCR is having more of an effect that can cause decreased oxidative and inflammatory response (Fig. 5A and C).

Taking our understandings from this study one step further, we examined gene expression findings outside the scope of our study in other publications from the NASA OSDR. In a 2019 study of space-flown mice, changes were reported to the retinal structure, oxidative stress, degradation of photoreceptors, and changes to circadian rhythms [18]. A specific technique of interest that was used in their study was X-ray micro-computed tomography (micro-CT), which images the ophthalmic structures. From this technique, they found decreased thickness to retinal structures and choroid. Another 2019 study published in the NASA OSDR observed changes to several retinal ganglion cell proteins (*Cryaa*, *Cryab*, *Cryga*, and *Cryba1*) in space flown mice [56]. This discrepancy could be attributed to the differences between simulated and actual spaceflight. Next, we reviewed experiments published in 2021 on mice that were also irradiated at BNL. These mice were exposed to GCRsim. Their findings showed that blood monocyte levels could be a promising biomarker for long-term spatial learning deficits in space [57]. The results from our study, and additionally the data from the OSDR, give credence to further investigate AG to evaluate if they aid mice molecularly to improve spatial learning.

The miRNAs for the AG in this study were let-7a-5p, miR-125b-5p, and miR-16-5p. These each have several downstream gene targets. For example, let-7a-5p targets *Myc* gene, and the proteins IL6, and TGFβ-R1 to name a few [[58], [59], [60]]. MiR-125b-5p influences MMP-13, I1β, and TNF-R2 proteins [61], [62]. MiR-16-5p targets MMP1 and MMP8 proteins and the IFN-γ gene [63], [64]. Between these three miRNAs there is a consistency for cytokines to be targeted, which we looked at when we evaluated *Il6* and *Il1β* in our gene expression. In *Il6*, we did not see consistent gene amplification and were not able to run statistical analysis. In *Il1β*, we did not see any significant trends between the experimental conditions. MMPs were also associated with two of these miRNAs, genomic results on MMPs were published on the OSDR in 2010. The authors used the Agilent whole genome 4 K × 44 K array system and found that MMPs 8, 12, and 13 had highly upregulated gene expressions in osteoclasts under simulated microgravity [65]. Additionally, in another study evaluating simulated microgravity versus 1G they saw that *Mmp9* had a log_2_ fold change > 1 [66].Thus showing the interaction of *Mmps* with simulated spaceflight and miRNAs. In addition, it has been shown that TGF-β cytokine was significantly increased in simulated microgravity. This was an interesting finding, proposing that TGFβ-R2 may have a role in osteoclast formation in microgravity [65]. From our brief comparison of our data to the NASA OSDR, it gave valuable insight into future directions for our study. While the scope of our study was focused on a small pre-selected cohort of genes, performing experiments on *in vitro* retinal ocular models, as well as *in vivo* experiments to determine retinal changes, would be valuable data to further determine the role of AG in the eyes under spaceflight simulations.

### 4.1 Limitations of the study

Naturally, there were limitations to this study. One limitation was in our validation of an AG treatment paradigm in mice. Previously, the scrambled AG and inhibition of our target miRNAs (let-7a-5p, miR-125b-5p, and miR-16-5p) were validated in 3D microvessel cell cultures [20], [22], [42]. We used the results from this study as confirmation that the AG were specific for these miRNAs. However, the scrambled AG and AG inhibition of let-7a-5p, miR-125b-5p, and miR-16-5p were not validated in our mouse eyes and spines. Another limitation was the lack of protein expression data. One mouse eye was used for gene expression data, and the contralateral eye was fixed in paraffin for sectioning and staining. Upon staining and imaging of the eyes, there were no discernible trends or patterns in the eye when evaluating DNA damage, mitochondrial, or apoptosis pathways. An additional limitation of this study was the lack of other ocular examinations, such as optical coherence tomography (OCT) or micro-CT of the mice while *in vivo*. During spaceflight, it has been established that there are choroidal and retinal folding that are appreciable on OCT imaging, along with decreased thickness as well [4], [18], [67]. It would be interesting to see if these structural changes are at all impacted by radiation alone or with radiation and microgravity. It would also be interesting to see if the AG had any impact on the structure of the retina, by OCT imaging. Further, this study was limited in sample size. In the current study, there was a large cost associated with the different arms of experimental paradigms involved. There was a sample size of 10 mice for each arm making a total of 120 female mice utilized for this experiment, however due to RNA quantity and outliers, not all samples amplified in each condition. This issue potentially led to a lack of statistical significance for certain genes. In the future, this study could be repeated with the same number or more mice to see if statistical significance could be reached in the genes that were profiled. An additional limitation of our study was the GCR model. The GCR was administered as a single dose that represents the GCR that would be experienced in totality by the astronauts. However, on actual missions, the astronauts would be exposed to a constant low-dose rate and mixed model of radiation over the course of the mission [68]. During which time, the DNA damage repair, oxidative stress, and inflammatory responses could vary on account of time for the body to readapt to the radiation-induced changes. A future direction to consider is remodeling administration of the GCR to a constant low-dose.

A future direction for SANS research is to specifically investigate oxidative stress pathways. In astronauts who developed SANS, there was a link between SANS symptoms and deficiencies in the one-carbon metabolism pathway [9], [11], [69]. In a cohort of astronauts, those who developed SANS, higher levels of homocysteine and cysteine were observed. Additionally, these astronauts were reported to have insufficient levels of B vitamins, specifically vitamin B12, which can then result in increased homocysteine and cysteine [9], [70], [71]. Elevated levels of these metabolites increase oxidative stress that can result in increased ROS. Increased stress pathways and ROS can be a driving factor of cellular damage, which can potentially lead to pathologies associated with SANS development. This is especially important in tissues like the retina, which is one of the most highly metabolic tissues in the body. Dysregulation of metabolic processes that result in retinal damage have also been documented in other optic neuropathies on Earth.

## 5 Conclusions

We identified that there is a need to elucidate the individual impacts from microgravity and deep-space radiation simulations to determine why there are differential effects observed with the combination of stressors. Overall, we saw that there were altered DNA damage repair, oxidative stress, and inflammatory pathways in AG-treated mice, which differed based on tissue type evaluated and the combination of simulated space stressors with AG. A future direction for this study is to evaluate the biochemical links between elevated ICP and oxidative stress in human ocular tissue, as well as understand the relationship between GCRs and oxidative stress in human ocular tissue.

## Author contributions

NEB performed methodology, investigation, data curation, formal analysis, validation, visualization and writing the original draft. RCM performed spinal tissue extraction and assisted with RNA extraction for all the samples. SSH performed the spinal tissue extraction and assisted with data analysis. AJK NGA, and LO assisted in methodology. RS assisted in methodology and manuscript review. SA performed eye extraction on the mice during the harvest. JCS and EAB were responsible for project design, manuscript review and approval. AB was responsible for funding acquisition, reviewing, and approving the manuscript. TPS was responsible for the conceptualization, project design, funding acquisition, project administration, supervision, reviewing and approving the final version of the manuscript.

## Ethics declaration

This study was conducted in accordance with the ARRIVE (Animal Research: Reporting of In Vivo Experiments) guidelines. This study was approved by the BNL IACUC (Approval No. 506).

## Declaration of generative AI and AI-assisted technologies

The authors do not have any declaration of generative AI and AI-assisted technology use.

## Declaration of competing interest

The authors declare the following financial interests/personal relationships which may be considered as potential competing interests:Tasneem Sharma reports financial support was provided by Indiana University School of Medicine. Afshin Behesti reports financial support was provided by NASA. Afshin Behesti reports financial support was provided by NASA Cooperative Agreement NNX16AO69A. Tasneem Sharma reports financial support was provided by Research to Prevent Blindness. Tasneem Sharma has patent #US11678658B2 issued to University of North Texas Health Science Center. Tasneem Sharma given her role as associate professor, had no involvement in the peer review of this article and had no access to information regarding its peer review. Full responsibility for the editorial process for this article was delegated to another journal editor. The other authors declare that they have no known competing financial interests or personal relationships that could have appeared to influence the work reported in this paper.

## Data Availability

Data will be made available on request.
